# Linking deep convection and phytoplankton blooms in the northern Labrador Sea in a changing climate

**DOI:** 10.1371/journal.pone.0191509

**Published:** 2018-01-25

**Authors:** Karthik Balaguru, Scott C. Doney, Laura Bianucci, Philip J. Rasch, L. Ruby Leung, Jin-Ho Yoon, Ivan D. Lima

**Affiliations:** 1 Marine Sciences Laboratory, Pacific Northwest National Laboratory, Seattle, WA, United States of America - 98109; 2 Atmospheric Sciences & Global Change Division, Pacific Northwest National Laboratory, Richland, WA, United States of America - 99354; 3 Now at Department of Environmental Sciences, University of Virginia, Charlottesville, VA, United States of America - 22904; 4 Woods Hole Oceanographic Institution, Woods Hole, MA, United States of America - 02543; 5 Now at Institute of Ocean Sciences, Fisheries and Oceans Canada, Sidney BC, Canada - V8L 4B2; 6 School of Earth Sciences and Environmental Engineering, Gwangju Institute of Science and Technology, Gwangju, Republic of Korea; Universidade de Aveiro, PORTUGAL

## Abstract

Wintertime convective mixing plays a pivotal role in the sub-polar North Atlantic spring phytoplankton blooms by favoring phytoplankton survival in the competition between light-dependent production and losses due to grazing and gravitational settling. We use satellite and ocean reanalyses to show that the area-averaged maximum winter mixed layer depth is positively correlated with April chlorophyll concentration in the northern Labrador Sea. A simple theoretical framework is developed to understand the relative roles of winter/spring convection and gravitational sedimentation in spring blooms in this region. Combining climate model simulations that project a weakening of wintertime Labrador Sea convection from Arctic sea ice melt with our framework suggests a potentially significant reduction in the initial fall phytoplankton population that survive the winter to seed the region’s spring bloom by the end of the 21^*st*^ century.

## Introduction

The formation of North Atlantic Deep Water (NADW) in the sub-polar North Atlantic, a key-component of the thermohaline circulation, plays a critical role in the climate of the earth system by driving the global ocean circulation and influencing poleward heat transport [1]. The NADW is comprised of three constituent water masses: 1) the Labrador Sea Water (LSW, forming the upper limb); 2) the Denmark Strait Overflow Water (DSOW, forming the bottom branch); and 3) the Iceland-Scotland Overflow Water (ISOW, sandwiched between them). While the latter two masses form in the Greenland and Norwegian Seas, the former originates in the Labrador Sea, which is the western branch of the sub-polar gyre of the North Atlantic [2]. Several studies suggest that climate change induced discharge of more freshwater from the Arctic may trigger a reduction of the NADW formation [3, 4], so it is important to understand its possible consequences. LSW formation by deep convection in the interior of the Labrador Sea is sensitive to surface forcing and lateral advection of freshwater from surface boundary currents that can alter vertical stratification. [3, 5, 6]. A decrease in the formation rate of the LSW may impact the North Atlantic gyre circulation [7] and the Atlantic Meridional Overturning Circulation [8]. As the physical consequences of a shutdown of convection in the Labrador Sea may be enormous, the ecological consequences that follow may also be significant.

The spring phytoplankton bloom in the North Atlantic stands out in global satellite ocean color data [9, 10]. Several recent studies hypothesize that convective mixing in the sub-polar North Atlantic plays a critical role in the timing and intensity of spring blooms in that region [9, 11, 12]. During dark winter months, multiple factors influence phytoplankton survival, e.g. photosynthetic growth (supported by nutrient-rich but relatively poor light conditions), zooplankton diapause, and phytoplankton losses due to zooplankton grazing, mortality and gravitational sinking. Seasonal deepening of the convective mixed layer during the fall and winter months may tilt the balance in favor of net positive phytoplankton growth by reducing grazing pressure via dilution of prey concentrations [9] and by re-entraining sinking phytoplankton [12], effectively converting the entire deep convective mixed layer into a virtual euphotic zone [11].

Convection plays another important role in the Labrador Sea spring bloom by influencing the plankton size distribution. In the context of global size-partitioned phytoplankton functional types, the contributions from nanoplankton (cell-diameter: 2-20*μ*m) and microplankton (cell-diameter: > 20*μ*m) jointly exceed 50% of the total phytoplankton carbon biomass in the high latitudes [13, 14]. While diatoms and dinoflagellates dominate the Labrador Sea spring bloom, colonial prymnesiophytes constitute the major early bloom species [15]. These larger plankton species sediment out of the fall mixed layer under their own weight and sink hundreds of meters into the deep ocean. However, a convection-induced rapid increase in the mixed layer depth (MLD) can entrain these sinking larger plankton, which would otherwise have escaped into the abyss, providing a pathway for facilitating sunlight-induced production and extending their residence time in the euphotic zone [12].

Few studies in the past have examined the relationship between MLD variability and plankton blooms in the Labrador Sea. Analysis of weekly MLD and surface chlorophyll concentration from SeaWiFS satellite data showed predominantly negative correlations poleward of 35°N-40°N in the Atlantic [16], with a couple of small regions of positive correlation, between 60°N-70°N to the west of Greenland in the Labrador Sea, and to the northeast of Iceland in the Greenland Sea. The interannual relationship between winter MLDs and depth-integrated chlorophyll inventory in the North Atlantic was recently examined [17], but the study did not include the Labrador Sea, a region of deep water formation. In this study, we extend that analysis and demonstrate the relationship between wintertime convection and the intensity of spring blooms in the Labrador Sea at interannual timescales and then employ a simple analytical model (Methods) to explore the role of plankton sedimentation; we then apply the analysis framework to climate model output to estimate the potential effects of climate change on the future of these blooms. The layout of the paper is as follows. In section 2, we describe the data, model and methods used in our analysis. The results are explained in section 3 and finally a discussion of the main conclusions and their implications is given in section 4.

## Materials and methods

### Observations

Data on phytoplankton biomass metrics for the northern Labrador Sea were collected from satellite remote sensing. We used surface chlorophyll-a concentration estimates from a merged satellite product developed by the European Space Agency’s Ocean Colour Climate Change Initiative (OC-CCI) to examine surface chlorophyll and to compute the depth-integrated chlorophyll inventory. This merged product has been developed using data from the following satellite sensors: SeaWiFS, MODIS-AQUA and MERIS. Version 3.1 of the dataset, available online from http://www.esa-oceancolour-cci.org/, was obtained for the 18-year period 1998-2015 at a horizontal spatial resolution of approximately 4 km. A comparison with single mission and other merged satellite ocean color products showed that the OC-CCI product provides more observations than other products, and is more consistent over time than other multi-mission products [18]. While monthly mean data were used to generate a climatology for climatological analysis, higher frequency 5-day mean data were used to examine interannual variability. Along with chlorophyll, we also obtained the number of chlorophyll observations to characterize uncertainty associated with the data. We generated a monthly climatology of the diffuse attenuation coefficient at 490 nm (K_490_), based on monthly mean estimates of (K_490_) from OC-CCI, and combined it with a climatology of Photosynthetically Available Radiation (PAR) based on SeaWIFS (obtained from http://mcc.jrc.ec.europa.eu/emis/) to compute the carbon-to-chlorophyll ratio (Θ).

Although several different ocean Mixed Layer Depth (MLD) products are available, we used MLD from two sources. For the climatological analysis, we used an observed MLD climatology from http://www.ifremer.fr/cerweb/deboyer/mld/Surface_Mixed_Layer_Depth.php. This climatological dataset, with a horizontal spatial resolution of 2°, was developed using nearly 880,000 *in situ* profiles of density from the World Ocean Database, World Ocean Circulation Experiment and Argo floats [19]. The climatological MLD data were used to compute the depth-integrated chlorophyll inventory and the carbon-to-chlorophyll ratio. On the other hand, to examine interannual relationships with surface chlorophyll concentration, we used 5-day mean MLD from SODA3 (Simple Ocean Data Assimilation—version 3.3.1) obtained from http://www.atmos.umd.edu/~ocean/. SODA3 eddy-permitting ocean reanalysis is produced using GFDL’s MOM5 ocean model forced by MERRA2 atmospheric reanalysis [20]. It has a spatial resolution of 0.25° near the equator, increasing to 0.1° near the poles. Data were obtained for the same 18-year period of 1998-2015. In both these products, the MLD is estimated as the depth where the potential density increases by 0.03 kg m^−3^ with respect to its value at the surface. Using *in situ* data from floats and Seagliders in the Labrador Sea, [21, 22] found that this definition of MLD reasonably represented the depth of active turbulent mixing or ‘mixing layer’ [23, 24] under a convective regime. The deep convection that results in deep wintertime mixing in the Labrador Sea, is not homegenous in time and tends to occur more episodically. High frequency variability in MLD may cause vertical gradients in phytoplankton concentration in the water column that could influence the depth-integrated net community production. Hence, to account for this, we use a MLD defined as above and high-frequency 5-day mean data when considering interannual relationships with surface chlorophyll.

Climatology of Euphotic Depth (ZEU), defined as the depth where the PAR reduces to 1% of its surface value, based on monthly mean data from the GlobColour project [25](available online at http://www.globcolour.info/), was used with climatological PAR to compute the depth-integrated chlorophyll inventory. For the observational analysis, the depth-integrated chlorophyll inventory is estimated as the product of surface chlorophyll concentration and the larger of the MLD and the Isolume Depth [26]. We assume a PAR value of 0.415 Ein m^−2^ day^−1^ as the threshold light level that can support photosynthesis [22, 26, 27]. With this assumption, following [26], the Isolume Depth is estimated as

$$\begin{array}{c} Isolume\;Depth=log\left(\frac{0.415}{0.98\;PAR}\right)\;\left(\frac{ZEU}{log(0.01)}\right) \end{array}$$

### Model

We used output from CESM 1.0 (Community Earth System Model version 1, [28])) to project changes in MLD for the Labrador Sea in a changing climate, following scenarios used in the fifth phase of the Coupled Model Intercomparison Project (CMIP5, [29]). Two climate change scenarios were considered: 1) an intermediate mitigations emission scenario typically identified as a “Representative Concentration Pathway” asymptoting to a 4.5 Wm^−2^ global-mean greenhouse gas forcing (RCP 4.5) and a higher emissions scenario asymptoting to an 8.5 Wm^−2^ global-mean greenhouse gas forcing (RCP 8.5) [30]. A three realization ensemble was used for each scenario to provide hints about variability in scenario projections. These model data are freely available from https://www.earthsystemgrid.org/. A mean MLD annual cycle was constructed for each decade to represent the MLD conditions for that decade. For consistency, the MLD was estimated using the same potential density criterion as was used for the observational analysis. Additionally, we obtained surface wind stress, sea surface temperature, sea surface salinity and precipitation data from the CESM model to understand the factors responsible for MLD changes projected by the model. While the surface wind stress data was used to examine the contribution of Ekman pumping, sea surface temperature and sea surface salinity data were used to understand changes in surface density. Further, precipitation data were used to evaluate the role of rainfall in surface salinity changes.

Measurements of plankton sinking velocities as a function of their cell sizes [31] and an analytical formulation [12] are used with CESM model output to develop a framework to characterize the fraction of fall phytoplankton population that remains in the mixed layer by the end of winter. The final phytoplankton population are estimated as a function of initial plankton population, changes in MLD and phytoplankton sinking rate for the idealized case of no net biological growth or loss over the winter. More specifically, the fraction of the fall mixed-layer phytoplankton population that survives the winter and remains above the depth of maximum winter mixing (*Fr*) is estimated as

$$\begin{array}{c} Fr=\prod_{i=1}^{6}fr_{i} \end{array}$$

where 1 corresponds to the month of October and 6 corresponds to the month of March, and *fr*_*i*_ is the fraction of phytoplankton population in month *i* − 1 that remains in the mixed layer by month *i*, given as

$$\begin{array}{c} fr_{i}=e^{\frac{W_{s}-{\frac{dH}{dT}}_{i}}{{\frac{dH}{dT}}_{i}}log\frac{H_{i}}{H_{i-1}}} \end{array}$$

Here *W*_*s*_ is the phytoplankton sinking rate, *H*_*i*−1_ and *H*_*i*_ are the MLDs for months *i* − 1 and *i* respectively, and ${\frac{dH}{dT}}_{i}$ is the rate of change of MLD between months *i* − 1 and *i*. A detailed derivation of these formulae is provided in S1 Text.

## Results and discussion

**Fig 1:**
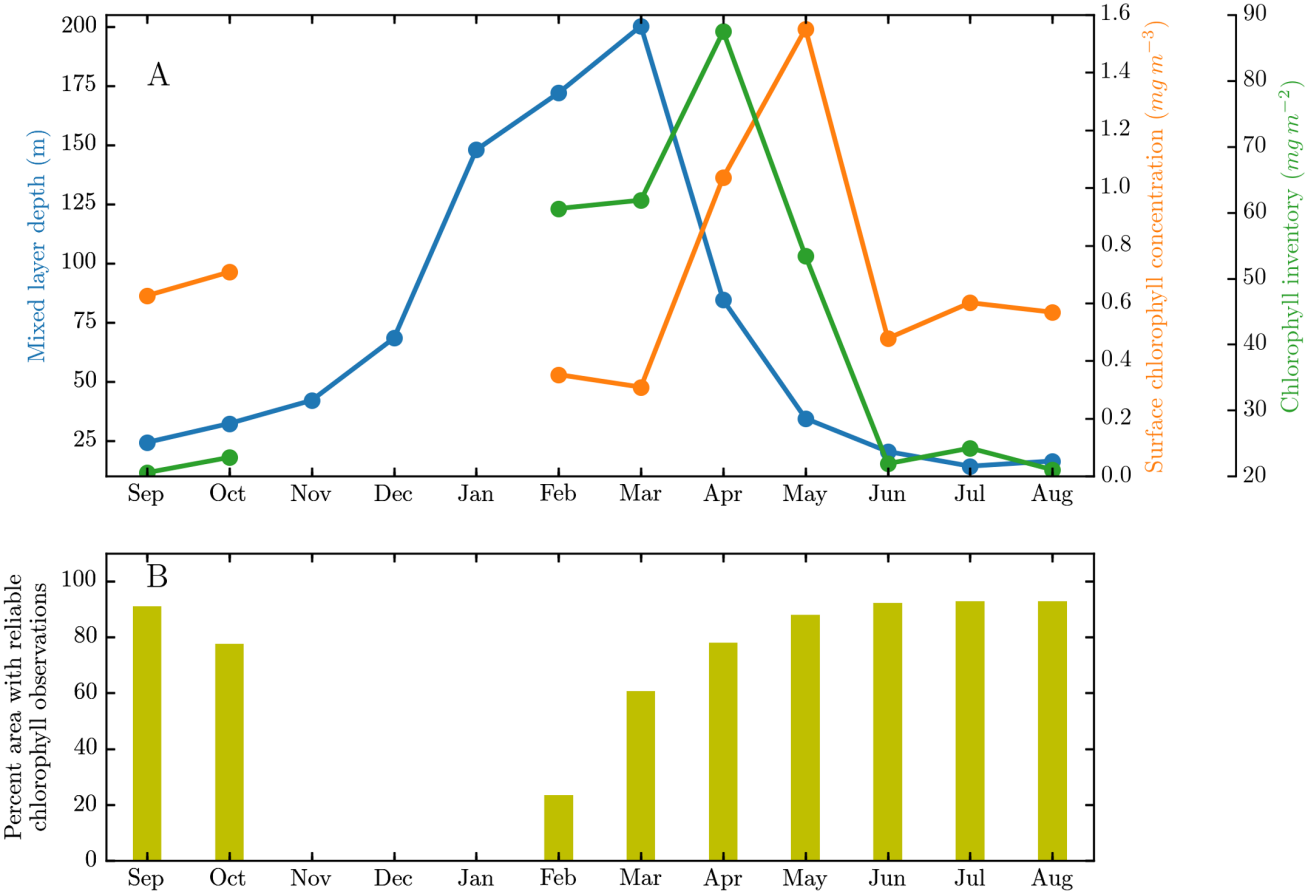
A) Climatological seasonal cycles of Mixed Layer Depth (MLD, m), surface chlorophyll concentration (mg m^−3^) and depth-integrated chlorophyll inventory (mg m^−2^), averaged over the northern Labrador Sea (60°W-50°W and 60°N-65°N) B) Average percent area of the northern Labrador Sea with satellite chlorophyll observations for each month.

We begin by examining the climatological seasonal cycles of MLD, surface chlorophyll concentration and depth-integrated chlorophyll inventory, averaged over the northern Labrador Sea between 60°W-50°W and 60°N-65°N, computed using reanalysis and satellite ocean color data (Fig 1A). A rapid increase in the mean MLD occurs during the winter months as a result of convective mixing in the Labrador Sea. While the mean MLD is about 70 m in December, it increases nearly by a factor of three to its maximum value of about 200 m in March. While the surface chlorophyll concentration increases sharply from March to April with a peak in May, the depth integrated chlorophyll inventory displays a peak in April, when MLD is shoaling but still relatively deep and chlorophyll concentrations are high. These signatures can be compared to those found previously in the sub-polar North Atlantic [17, 26]. The timing of the peak spring bloom in surface concentration and depth-integrated inventory is similar to the sub-polar pattern, where the inventory maximum typically precedes the concentration maximum [17, 26]. The close timing of the observed physical and biological seasonal cycles are suggestive of the importance of the over-wintering phytoplankton population, and associated governing mechanisms, on the subsequent spring bloom. Note that gaps in surface chlorophyll concentration and depth-integrated chlorophyll inventories in November, December and January arise because satellite retrieval of ocean color are unavailable during low light conditions at high latitudes during those months. This is illustrated in Fig 1B that shows the average percent area of the northern Labrador Sea with satellite surface chlorophyll measurements. During the months of November, December and January, less than 1% of the area has reliable observations of surface chlorophyll.

**Fig 2:**
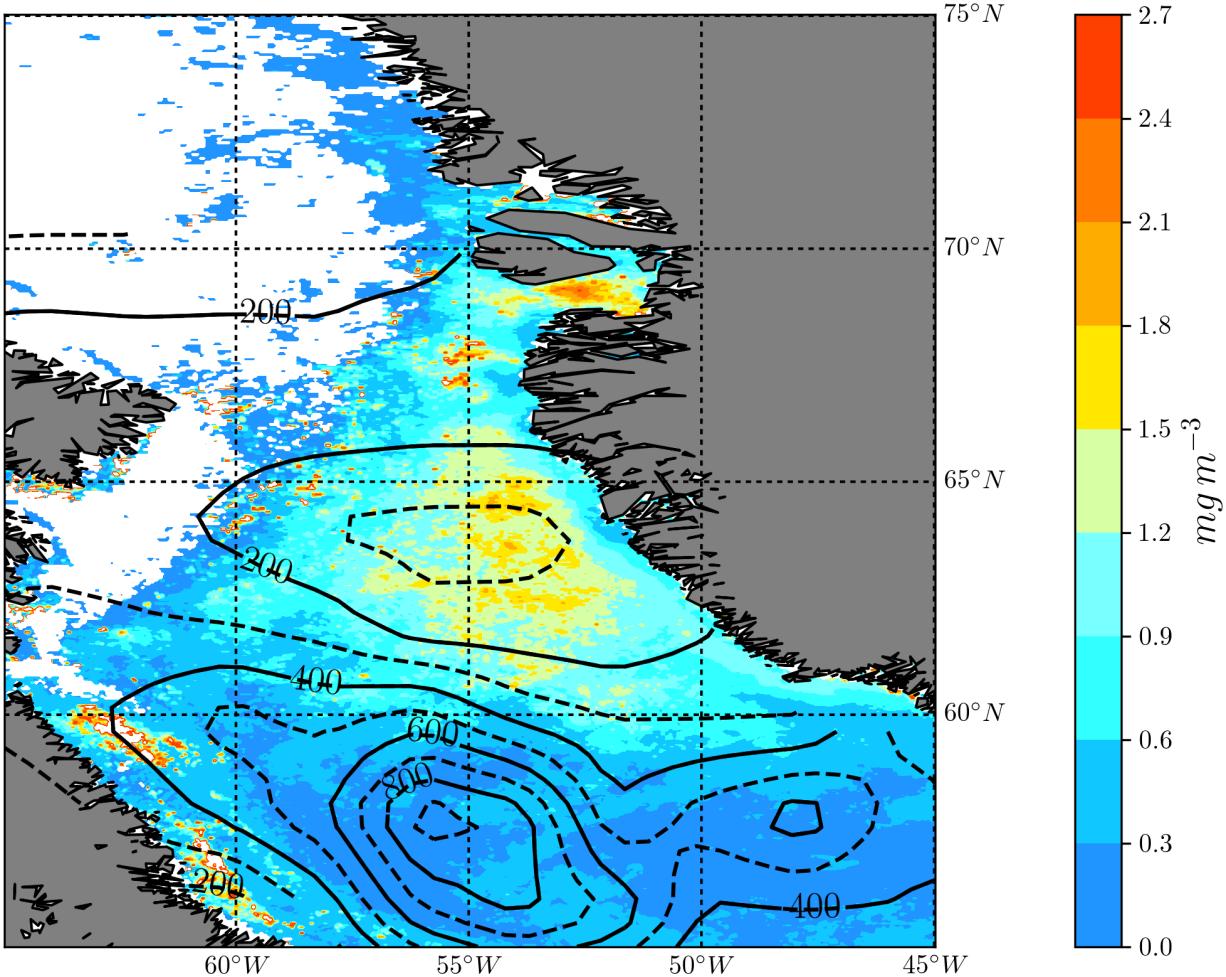
Climatological April surface chlorophyll concentration (mg m^−3^) with contours of climatological March MLD (m) overlaid. The contour interval is 100 m with solid lines used for labelled contours.

The spatial distribution of climatological April surface chlorophyll concentration is shown in Fig 2 with the contours of the climatological March MLD overlaid. The combination of the ocean color and MLD data shows a distinct region, situated between Greenland and Canada, where the levels of chlorophyll are regionally high. This patch of high chlorophyll concentration corresponds to the Labrador Sea spring phytoplankton bloom, which is a part of the North Atlantic bloom. This bloom has two distinct phases in the Labrador Sea of which the north bloom, which occurs to the north of 60°N during May-June (indicated by the rectangular box), is the dominant one [32] and produces the greatest quantity of zooplankton biomass [33]. The secondary bloom occurs in the central Labrador Sea during June-July. The intensity and timing of spring blooms in the northern Labrador Sea are believed to be partly dependent on the strong eddy activity in this region [34]. Eddies in the region, which form due to baroclinic instability of the West Greenland and Irminger Current Systems and instabilities of the Boundary Current System [35] advect freshwater offshore from the West Greenland Current, leading to upper-ocean stratification and consequently influence the spring phytoplankton bloom [34].

**Fig 3:**
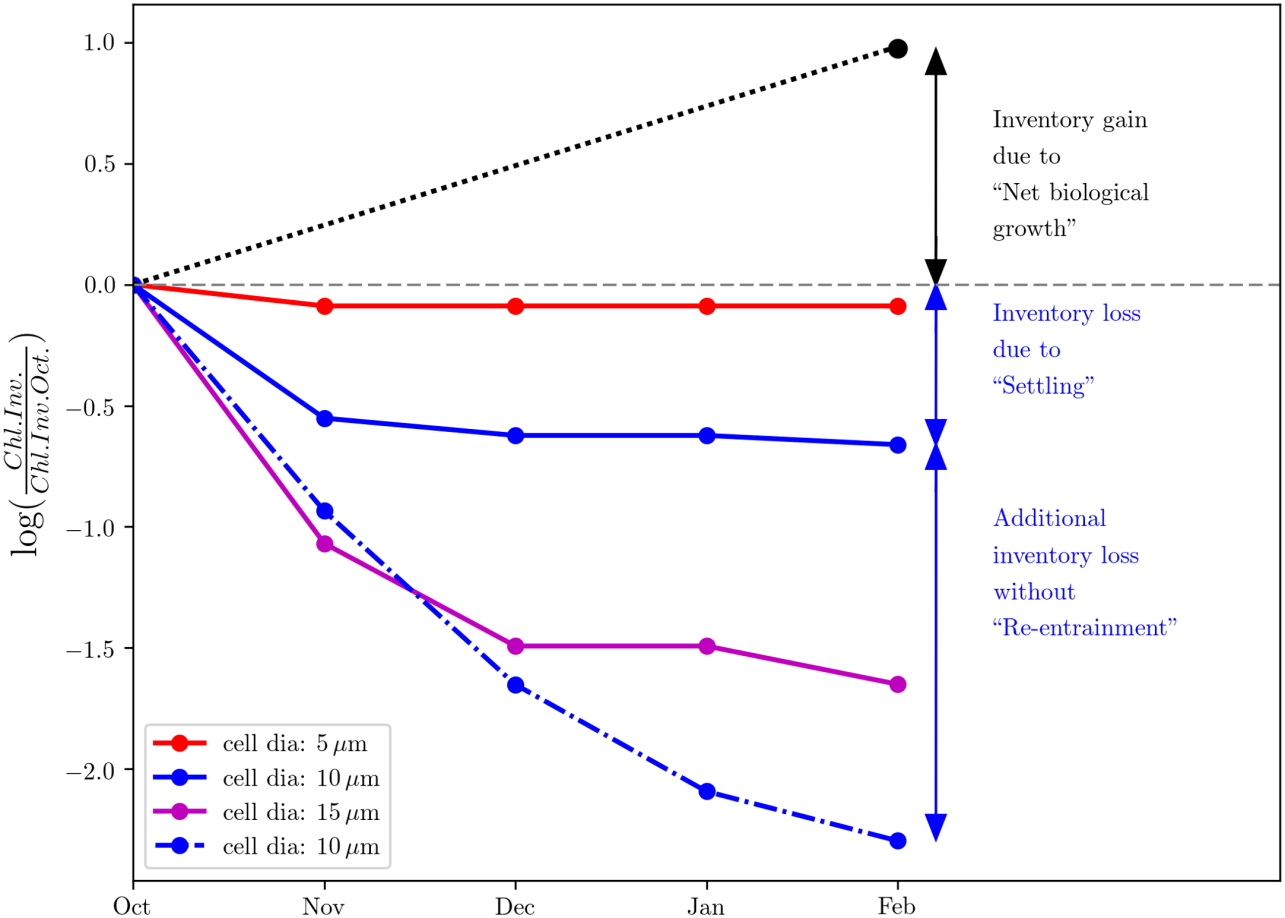
The black dotted-line represents the climatological observed change in chlorophyll inventory between October and February, based on the seasonal cycle shown in Fig 1A. The three solid curves indicate changes in senescent plankton population with time for various cell sizes (red, blue and magenta for 5, 10 and 15 *μ*m respectively) with sinking as well as re-entrainment considered. The blue dashed-line indicates changes in plankton population with time for a plankton size of 10 *μ*m with sinking only and without re-entrainment.

An interesting feature of Fig 1A is the change in surface chlorophyll concentration and chlorophyll inventory between October and February. While the surface chlorophyll concentration decreases from October to February, the depth-integrated chlorophyll inventory increases. The black dotted-line in Fig 3 shows that the chlorophyll inventory increases by a factor of about 2.5 over the course of winter, indicating that there is a substantial net biological growth during this period, which may reflect reduced grazing because of dilution. Also shown in Fig 3 is the temporal evolution of the mixed layer chlorophyll inventory due to sinking of senescent phytoplankton cells for different plankton cell diameters, based on the analytical model described in Methods. These terms represent plankton losses as a result of gravitational settling; larger cells sediment more rapidly following Stoke’s Law, even for the same cell density anomaly relative to seawater [36]. The larger-diameter plankton settle out faster from the mixed layer relative to the smaller-diameter plankton and the reduction in their inventory with respect to their initial population is relatively larger. The observed net growth of the population (black dotted-line, Fig 3) implies that the effects of gravitational sinking are more than compensated for by net biological growth for a plankton size between 10 and 15 *μ*m. However, the substantially larger losses in plankton population without re-entrainment, even for a plankton cell size of 10 *μ*m, underlines the significance of the process. Although a more complete analysis would also account for photosynthetic growth, zooplankton grazing and other loss and mortality terms [17], a simple scale analysis suggests that the effects of re-entrainment on plankton population can be significant, particularly for the larger plankton (S1 Text). Note that while we have not accounted for effects of photoacclimation on chlorophyll explicitly, a computation of the climatological seasonal cycle of the carbon-to-chlorophyll ratio indicates that it might be reasonable to assume it to be a constant for the fall and winter months, the period of interest in our study (S1 Text). Additional complexity could also arise because some non-motile phytoplankton cells (including some diatoms) are able to adjust their buoyancy and thus sinking speeds, at least over relatively short time periods and within some range governed by metabolic constraints [37, 38].

The MLD contours in Fig 2 indicate that the principal site of deep convection is located near 56°W and 58°N, where the maximum MLD exceeds 800 m. The presence of strong eddies in the northern Labrador Sea act to restratify the water-column and to reduce the convective activity relative to the central Labrador Sea [35]. Despite this, mean MLD values exceeding 200 m suggest that weaker convection is also present in the northern Labrador Sea region. As previously noted, recent observations suggest that deep convection may aid the formation of plankton blooms through entrainment of heavier and larger plankton species, such as diatoms, back into the convective mixed layer [11, 12]. Thus, size-differentiated effects of winter zooplankton grazing would also influence both phytoplankton population and community composition.

**Fig 4:**
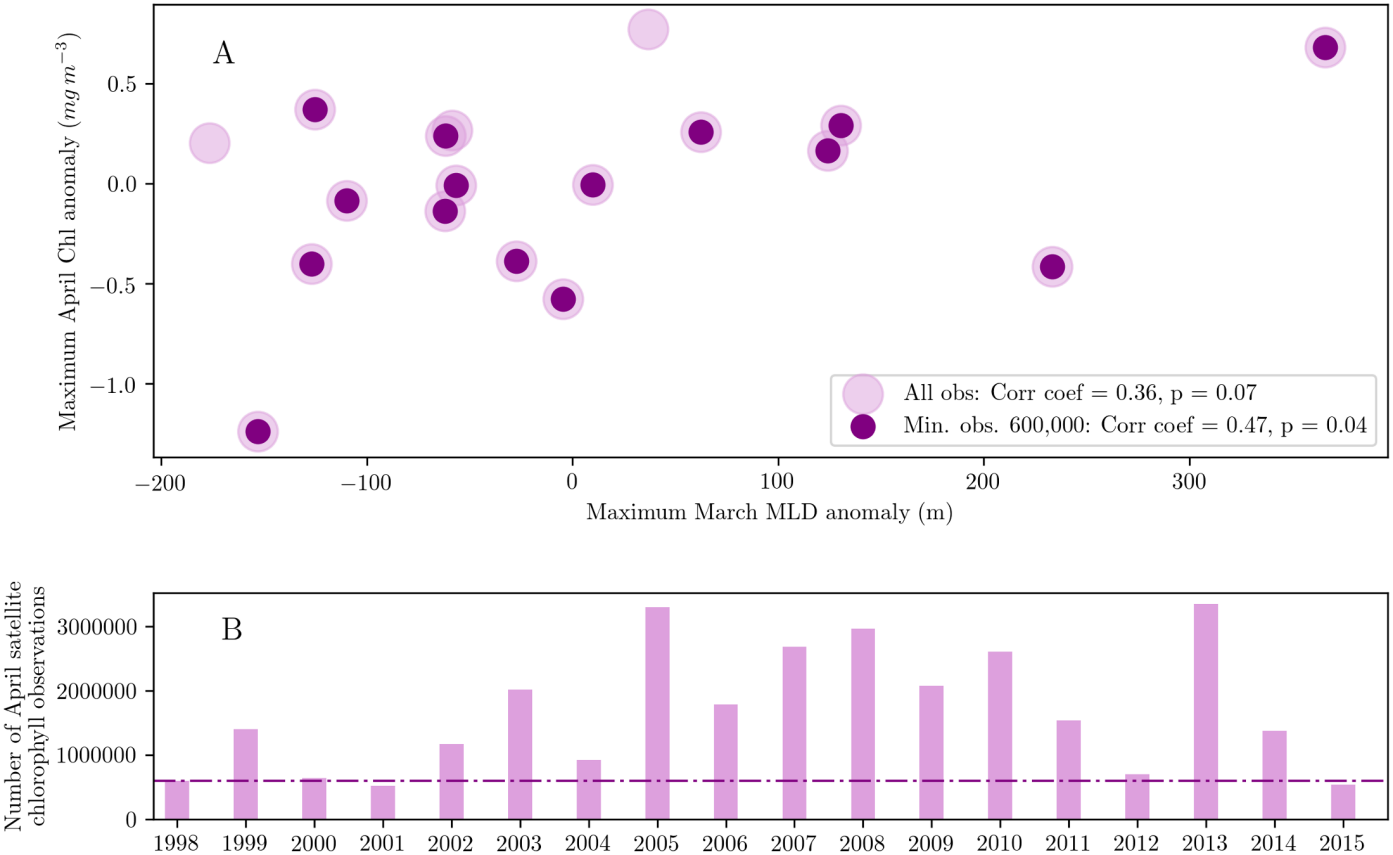
A) Maximum March MLD anomalies (m) plotted against maximum April surface chlorophyll concentration anomalies (mg m^−3^), averaged over the northern Labrador Sea and for the period 1998-2015 (in pink). Purple dots centered on pink dots indicate data points with atleast 600,000 chlorophyll observations in the northern Labrador Sea. B) Number of April satellite chlorophyll observations for each year in the northern Labrador Sea. The dash-dotted horizontal line indicates the cut-off level of 600,000 chlorophyll observations used in A.

Wintertime deep convection in the northern Labrador Sea could also play a role in the spring bloom at interannual timescales. Evidence to support this hypothesis is presented in Fig 4A that shows the maximum April surface chlorophyll anomaly as a function of the maximum March MLD anomaly, a proxy for convective mixing. Both quantities are averaged over the northern Labrador Sea (60°W-50°W and 60°N-65°N). Maximum April surface chlorophyll is well-correlated with the maximum March MLD, with a correlation coefficient of 0.36, statistically significant at the 90% level based on a Student’s t-test. Fig 4B shows the number of April satellite chlorophyll observations in the northern Labrador Sea for various years. It is clear that satellite coverage varies substantially from year to year. Considering that the area of the northern Labrador Sea is nearly 0.3 million square km, and that the resolution of data is about 4 km, about 600,000 observations of surface chlorophyll are needed per month in the northern Labrador Sea to obtain an average of approximately one observation per bin per day. If we subsample chlorophyll data using this threshold of 600,000 observations, we eliminate 3 years of data out of 18. Two out of these three years, 1998 and 2001, occur during the period prior to 2003 when SeaWIFS was the only sensor available. The third year with poor data is surprisingly 2015. Although establishing the cause of this is beyond the scope of this paper, we speculate that it may be related to the reduced quality of MODIS-Aqua data beyond 2014 (http://esa-oceancolour-cci.org/?q=node/184). When these three years are removed, the correlation between maximum April surface chlorophyll and the maximum March MLD anomalies increases to 0.47, a value significant at the 95% level. Peak surface chlorophyll values typically occur in April, a month after the deepest seasonal MLD in March. There is a strong positive correlation between the year to year anomalies of March MLD and April surface chlorophyll the following month. This correlation indicates that deeper winter mixed layers enhance wintertime surface accumulation rate, and the magnitude of the subsequent surface spring bloom, likely reflecting lower wintertime loss rates from some combination of re-entrainment of sinking cells and the ‘dilution—re-coupling’ hypothesis. A deeper MLD in winter may also promote a stronger spring bloom through enhanced nutrient entrainment. This analysis supports the argument that convective mixing plays an important role in the interannual variability of Labrador Sea plankton blooms and leads us to consider the implications of this relationship in the future.

Mounting evidence clearly shows that the Arctic sea ice cover is declining as a consequence of climate change with the potential for ice-free Arctic summers in the not too distant future [39–41]. Satellite data shows that the Arctic ice cover has been decreasing at a rate of nearly 0.44 million km^2^ per decade since 1978 [41] with similar evidence for decreases in the thickness of the ice-cover, indicating a dramatic decrease in the Arctic sea-ice volume. Climate change is also projected to enhance melting of the Greenland ice sheet and increase riverine discharge into the Arctic [42]. Since deep convection in the Labrador Sea and the associated LSW formation is sensitive to Arctic freshwater discharge, rapid changes in the Arctic cryosphere may have consequences for the spring blooms of the northern Labrador Sea.

We consider consequences of this possibility using output from a fully-coupled climate model and an analytical model (Methods) to estimate potential changes in phytoplankton spring bloom due to those in wintertime convection in the Labrador Sea. The CESM is able to accurately simulate the location of deep water formation in the sub-polar North Atlantic [43]. The mean MLD conditions are similar to observed, indicating that the model has a realistic representation of convective activity in the North Atlantic. The site of active convection is located in the central Labrador Sea and to the southwest of Cape Farewell, the southern tip of Greenland, and is in good agreement with observations [43]. Further, a comparison of 23 CMIP5 climate models shows that the CESM is one of the few models that convects correctly in the Labrador Sea and in the northern part of the North Atlantic sub-polar gyre, albeit strongly [44]. Also, the model is able to realistically represent changes in Arctic sea ice [45], making it an appropriate model for this study. Considering the changes in Arctic sea ice predicted by an ensemble of CESM simulations under the RCP 4.5 scenario, we find that the model projects a rapid decrease in the areal extent of Arctic sea ice over the next few decades (S1 Fig). The results are even more dramatic for summer Arctic ice [46]. From the current value of nearly 6 million km^2^, the September Arctic ice area is projected to drop by 50% by the year 2100. In the case of RCP 8.5 scenario, the reduction in Arctic ice nearly follows that in RCP 4.5 until 2040, but occurs more rapidly between 2040 and 2100, reaching near ice-free summer Arctic conditions by 2080 [46].

The above mentioned changes in Arctic sea ice cover, and consequent freshwater export [47], can have a substantial impact on wintertime deep convection in the Labrador Sea [46]. To understand this, we examine changes in MLD, a proxy for convective mixing, in the northern Labrador Sea (S2 Fig). Model results show that the winter mean MLD may progressively decrease suggesting a projected decline in wintertime convection. By 2050, the maximum winter MLD averaged over the northern Labrador Sea may reduce by nearly 40% under the RCP 4.5 and 8.5 scenarios respectively. Furthermore, by 2070, the maximum winter MLD averaged over the northern Labrador Sea may reduce by about 60% and 80% under the RCP 4.5 and 8.5 scenarios respectively. The main source of freshwater for the Labrador Sea is the East Greenland Current [48], which is fed by low saline waters from the Arctic through the Fram Strait [49]. Thus, changes in Labrador Sea MLD can occur due to enhanced freshwater flux through the Fram Strait brought about by Arctic sea ice melt [47]. An analysis of various factors that contribute to stratification in the Labrador Sea indeed suggests that changes in freshwater flux are the likely cause for the projected reduction in wintertime MLD (S1 text and S3 Fig). While these changes in MLD were based on output from CESM, analysis of MLD changes from 23 other CMIP5 climate models reveals that most models predict a weakening of convective activity in the Labrador Sea under climate change, suggesting that changes in CESM are consistent with most climate models [50].

**Fig 5:**
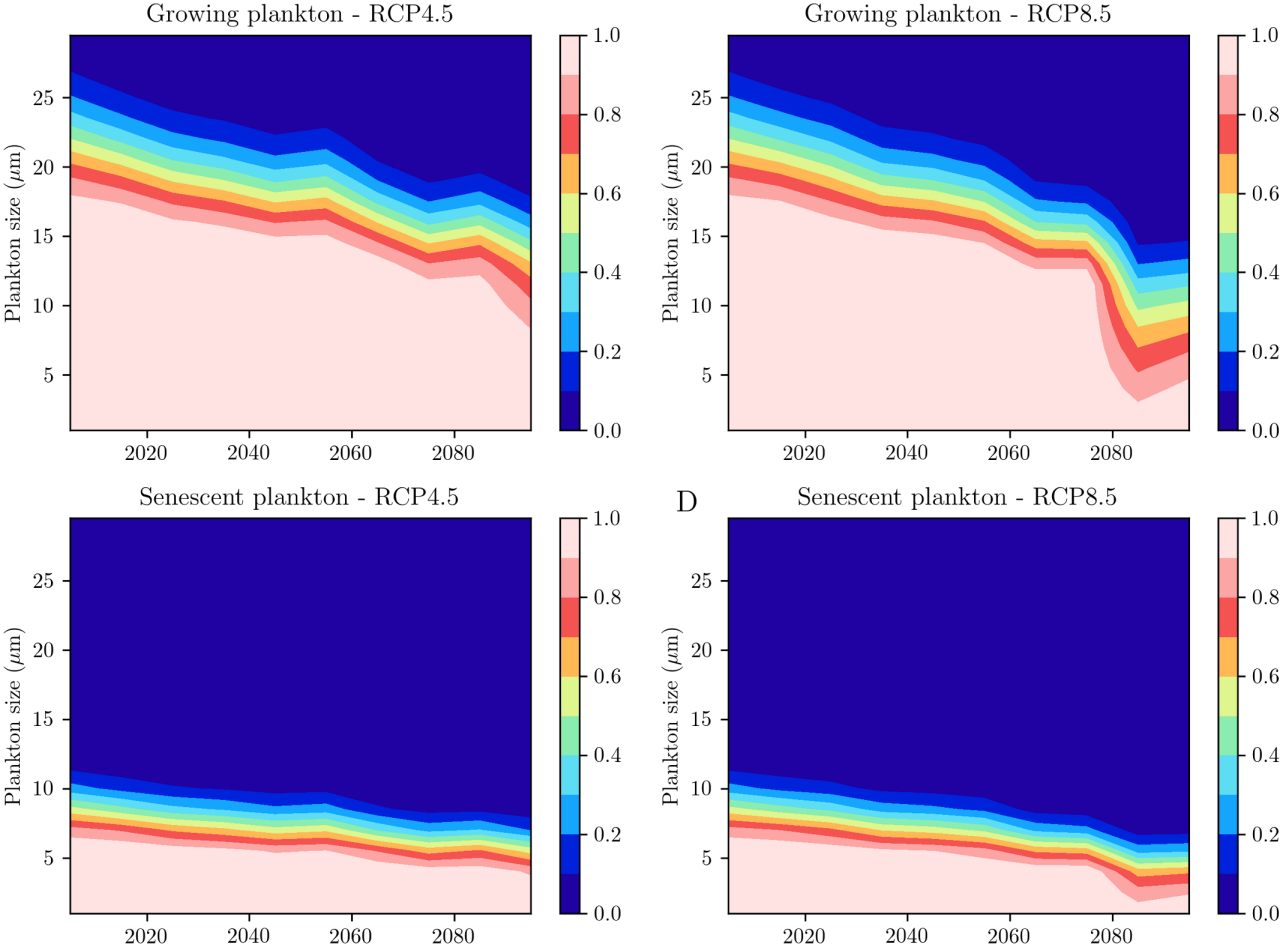
Projected fraction of growing fall phytoplankton population that survives the winter, as a function of plankton cell-diameter in A: RCP 4.5 scenario B: RCP 8.5 scenario. C: and D: As in A and B but for senescent phytoplankton.

Finally, we examine the impact of the projected MLD changes on Labrador Sea spring blooms. To this end, we combine the MLD projections of the CESM model with an analytical model (Methods) to estimate the effects of gravitational sinking and mixed-layer re-entrainment on the fraction of fall phytoplankton population that survives the winter and acts as seed for the spring bloom (Fig 5). As the plankton cell-diameter increases, cells sediment out of the mixed layer more rapidly, and fewer large phytoplankton survive the winter. Thus, nanoplankton and microplankton with cell-diameter greater than 10 *μ*m (phytoplankton species contributing majorly to the spring bloom), are more sensitive to changes in convective activity than picoplankton (phytoplankton species with cell-diameter less than 2 *μ*m). For growing phytoplankton under present climate conditions, only plankton with cell diameter less than 25 *μ*m survive the winter. While the deepening MLD is able to retain nearly 80% of the plankton under 10 *μ*m, the larger plankton experience losses ranging between 20-80% (Fig 5A). A significant fraction of plankton population with cell diameter below 10 *μ*m would be retained under the RCP 4.5 scenario. However, losses for phytoplankton with cell diameter between 10 *μ*m and 25 *μ*m would range between 10% and 90% (Fig 5A). Under the RCP 8.5 scenario, even plankton with cell diameter under 10 *μ*m would experience substantial losses (Fig 5B). Another interesting feature to note is that while the fraction of fall plankton population that survives the winter undergoes a steady decline under the RCP 4.5 scenario, it experiences a period of sharp decrease at around 2080 under the RCP 8.5 scenario. These aspects are consistent with changes in MLD in the respective scenarios. In comparison to growing plankton, senescent cells, which sink relatively faster, experience a relatively higher loss of the initial phytoplankton population (Fig 5C and 5D). Senescent plankton with cell diameter larger than 10 *μ*m sediment out of the deepening fall mixed layer under current climate conditions, while losses ranging between 10-90% occur for nanoplankton with cell diameters of about 5-10 *μ*m. Under future climate conditions, the loss of senescent fall phytoplankton population from the mixed layer would increase significantly, with even picoplankton experiencing losses of nearly 20% under the RCP 8.5 scenario (Fig 5D).

## Conclusions

Footprints of anthropogenic climate change are clearly discernible in large marine eco-systems surrounding the Labrador Sea region [51]. Decreases in the summer Arctic sea ice cover are changing the surface stability and buoyancy flux, leading to the real possibility that wintertime convection may be substantially reduced in the Labrador Sea over the next few decades. While convection is primarily a vertical process, past studies suggest that changes in freshwater transport and lateral circulation also act to stratify water column and disrupt convection in the Labrador Sea, impacting the marine ecosystems of that region. For instance, a reduction in the convection may change the Labrador Current, which may then impact the marine ecosystems and seabird populations of the Labrador Sea region [5]. Changes in convection may also influence the Greenland Current and consequently the biological activity near the Ammassalik Front off east Greenland Shelf [52].

Previous studies have examined the role of convective mixing in spring blooms of the North Atlantic, but their analysis did not include the Labrador Sea. While evidence of living phytoplankton in deep wintertime convective mixed layers was previously presented based on ship cruise data from the Norwegian Sea [11], wintertime vertical profiles of phytoplankton obtained from an autonomous float in the western subpolar North Atlantic [53], and bio-Argo floats in the Labrador Sea suggests that similar processes may also be happening in that region. For instance, see http://www.oao.obs-vlfr.fr/bioargo/PHP/lovbio044b/lovbio044b_105_00_09.txt.jpeg for a profile from lovbio044b, a float that was referenced in [54] or http://www.oao.obs-vlfr.fr/bioargo/PHP/lovbio032b/lovbio032b_112_00_09.txt.jpeg for a profile from float lovbio032b. In this study, we show that vertical mixing processes associated with wintertime convection may directly influence the marine ecosystems by playing a critical role in the spring phytoplankton blooms of the Labrador Sea. We demonstrate that the maximum winter MLD, a proxy for convection, is positively correlated with the April surface chlorophyll concentration in the northern Labrador Sea at interannual timescales. However, the tight observed interannual relationship between deepest winter MLD values in March and peak surface chlorophyll values a month later in April, probably arises from several mechanisms playing complimentary roles.

As convective activity deepens the winter mixed layer, the resultant dilution of phytoplankton population reduces the chance of predator-prey encounters that can occur between phytoplankton and zooplankton, which in turn can lead to a decrease in phytoplankton losses through grazing [11]. Later, when spring arrives, warmer conditions stratify the upper-ocean and lead to a spring bloom despite a re-coupling of the interaction between phytoplankton and zooplankton [9]. A few recent studies have suggested that this ‘dilution—re-coupling’ hypothesis may be responsible for the positive correlation between maximum winter MLD and depth-integrated chlorophyll inventory in the sub-polar North Atlantic [17]. Also, according to the ‘disturbance-recovery’ hypothesis [55], mixed layer deepening and dilution also act to decrease both biological growth and loss rates, with a larger effect on loss rate leading to net biomass accumulation during late fall and winter prior to the initiation of the surface Spring bloom. In this study, we primarily focused on an other factor, the ability of convection to entrain larger phytoplankton that would have otherwise sedimented out of the mixed layer (see scaling exercise including net biological terms in Supporting Information section 2). The relative importance of these different mechanisms needs to be established and it remains an area for future work.

We have used a relatively simple analytical model to estimate the effects of gravitational sinking and re-entrainment on the future of spring blooms in the Labrador Sea based on projected changes in the MLD from a fully-coupled climate model. Though this can provide valuable insights into the future of Labrador Sea spring blooms, in reality there are other factors, such as lateral circulation and the availability of nutrients and light, that also change with time [56]. For example, the marine ecosystem models included in CMIP5 exhibit substantial reductions in net primary production over much of the sub-polar North Atlantic associated with increased vertical stratification and lower nutrient supply [57, 58]. Feedbacks with zooplankton biomass and grazing rates also need to be considered. Further studies with wintertime field observations and more sophisticated models that have a more realistic representation of plankton dynamics are needed to better understand the effects of seasonal convection and climate change on regional plankton blooms.

## Supporting information

S1 Text Derivation of the analytical model to estimate the fraction of the fall phytoplankton population that survives the winter, Analysis revealing the relative significance of plankton re-entrainment, Estimating the impact of photoacclimation on carbon-to-chlorophyll ratio and Analysis of various factors contributing to future stratification changes in the northern Labrador Sea. (PDF)

S1 Fig Projected changes in Arctic ice. A: Mean ice-fraction averaged over the years 1990-2005. Mean ice-fraction projected under the RCP 4.5 scenario averaged over the years B: 2020-2029 C: 2030-2039 D: 2040-2049.
(PDF)

S2 Fig Projected changes in the northern Labrador Sea winter MLD. Projected changes in March MLD (m) conditions in the northern Labrador Sea under the RCP 4.5 (purple) and the RCP 8.5 (yellow) scenarios. The ensemble mean is indicated by the thick line, while the shading around the line represents the spread among the ensemble members.
(PNG)

S3 Fig Analysis of various factors contributing to future winter stratification changes in the northern Labrador Sea. Time series of A: Ekman pumping (m s^−1^), B: Surface density (kg m^−3^), and C: Precipitation (kg m^−2^s^−1^), for the month of March and averaged over the northern Labrador Sea under the RCP 4.5 scenario. In panel B, the black curve represents density when both temperature as well as salinity are changing, the blue curve represents density with sea surface salinity (sss) fixed at its initial value and the red curve represents density with sea surface temperature (sst) fixed at its initial value. The trend values indicated are statistically significant at the 95% level.
(EPS)
