# Supplementary material for: Linking deep convection and phytoplankton blooms in the northern Labrador Sea in a changing climate
Source: PLoS One. 2018 Jan 25;13(1):e0191509. doi: 10.1371/journal.pone.0191509 (PMC5784959; doi:10.1371/journal.pone.0191509)
Supplement: S1 Text — (PDF) [file pone.0191509.s001.pdf]

Linking deep convection and phytoplankton blooms in the northern Labrador Sea in a changing climate

Karthik Balaguru[1,2,*], Scott C. Doney[3,4], Laura Bianucci[5,1], Philip J. Rasch[2], L. Ruby Leung[2], Jin-Ho Yoon[6], Ivan D. Lima[4]

1 Marine Sciences Laboratory, Pacific Northwest National Laboratory, Seattle, WA, USA - 98109.
2 Atmospheric Sciences & Global Change Division, Pacific Northwest National Laboratory, Richland, WA, USA - 99354.
3 Now at Department of Environmental Sciences, University of Virginia, Charlottesville, VA, USA - 22904.
4 Woods Hole Oceanographic Institution, Woods Hole, MA, USA - 02543.
5 Now at Institute of Ocean Sciences, Fisheries and Oceans Canada, Sidney BC, Canada - V8L 4B2.
6 School of Earth Sciences and Environmental Engineering, Gwangju Institute of Science and Technology, Gwangju, Republic of Korea.

* karthik.balaguru@pnnl.gov

Supporting Information

Analytical model

The phytoplankton depth-integrated biomass or population P (g m^{-2}) is given by the vertical integral of concentration C (g m^{-3}) over depth from the surface to the mixed layer depth H

$$P = \int_0^H C dz = CH \quad \text{(SI1)}$$

which reduces to the term CH assuming a homogenous mixed layer. Since the plankton population can either change due to net growth/loss or entrainment/detrainment, the rate of change of phytoplankton population in the mixed layer can be written as

$$\frac{dP}{dt} = H\frac{dC}{dt} + C\frac{dH}{dt} \quad \text{(SI2)}$$

The first term on the RHS represents the change in phytoplankton population within the mixed layer due to a change in concentration caused by a net biological growth or loss and gravitational settling. The second term represents the change in plankton population due to entrainment/detrainment from variations in the mixed layer depth or the control volume. Note that while the phytoplankton concentration corresponding to the first term on the RHS is the mixed layer concentration, the plankton concentration corresponding to the second RHS term depends on the sign of $\frac{dH}{dt}$. When the mixed layer is deepening ($\frac{dH}{dt} > 0$), it is the concentration right below the mixed layer and when the mixed layer is shoaling ($\frac{dH}{dt} < 0$), it is the mixed layer plankton concentration. To allow for re-entrainment of sinking cells, the phytoplankton concentration just below the mixed layer must be non-zero. For simplicity, in this study,

we assume that the concentration just below the mixed layer is determined solely by sinking from above. Thus, the plankton concentration within the mixed layer and just beneath the mixed layer are set as equivalent. This is in contrast to studies that focus on the "dilution-recoupling hypothesis" where the sub-mixed layer concentration is often set to zero and there is no consideration of re-entrainment [1].

Now, if the phytoplankton sinking speed is W_s, the rate of change in plankton population due to the sinking flux at the base of the mixed layer can be written as the product of W_s and C for a given mixed layer depth. Using this in SI2, the rate of change of plankton population may be written as

$$\frac{dP}{dt} = -W_s C + C\frac{dH}{dt} = -W_{eff} C = -W_{eff}\frac{P}{H} \quad \text{(SI3)}$$

where W_{eff} is defined as the effective phytoplankton sinking speed ($W_s - \frac{dH}{dt}$), which includes the effects of gravitational sinking of plankton as well as entrainment/detrainment. If the rate of change of mixed layer depth is assumed constant over a given period, the above equation when integrated leads to the framework of [2]

$$P_t = P_i\, e^{\int_0^t \frac{-W_{eff}}{H_t} dt} \quad \text{(SI4)}$$

Here P_i is the initial phytoplankton population, P_t is the phytoplankton population at time t and H_t is the mixed layer depth at time t. Thus, the fraction of initial plankton population that remains in the mixed layer (fr) at time t can be written as

$$fr = \frac{P_t}{P_i} = e^{\int_0^t \frac{-W_{eff}}{H_t} dt} \quad \text{(SI5)}$$

During the fall and winter months when the mixed layer depth is increasing ($\frac{dH}{dt} > 0$), it is often assumed that particles that sink below the deepening mixed layer are lost from the system. However, when the sinking velocity for a particle size class is less than or equal to the mixed layer deepening rate averaged over some time period ($W_s \le \frac{dH}{dt}$), then no particles of this size class are lost from the system.

From [3], the plankton sinking rate W_s (m day^{-1}) as a function of the cell-diameter cd (μm) for growing cells can be written as

$$W_s = 0.023\, cd^{1.12} \quad \text{(SI6)}$$

and for senescent cells as

$$W_s = 0.062\, cd^{1.21} \quad \text{(SI7)}$$

Next, if we assume a linear rate of change in mixed layer depths k between two successive months and if the initial mixed layer depth is H_i, then the mixed layer H_t at time t can be written as

$$H_t = H_i + kt \quad \text{(SI8)}$$

If we assume that the plankton settling rate 'W_s' is only a function of the cell diameter (cd) and that the other factors that influence it, such as the viscosity (ν), are time invariant, then equation SI5 can be evaluated between months 'i-1' and 'i' as

$$fr_i = e^{\frac{-W_{eff}}{k_i} log \frac{H_i}{H_{i-1}}} \quad \text{(SI9)}$$

Here fr_i is the fraction of phytoplankton population in month $i-1$ that remains in the mixed layer by month i, k_i is the rate of change of mixed layer depth between months $i-1$ and i, H_{i-1} is the MLD in month $i-1$ and H_i is the MLD in month i. This fraction is evaluated for each successive pair of months from September to March.

Note that by assuming a linear rate of change of MLD between any two months, W_{eff} becomes time-invariant in the integral shown in equation SI5. Then, the fraction of plankton population in September that remains in the mixed layer by March (Fr) is given by the product of the fractions.

$$Fr = \prod_{i=1}^{6} fr_i \tag{SI10}$$

where 1 corresponds to the month of October and 6 corresponds to the month of March. Note that our model does not account for increased mortality of phytoplankton cells retained in a deepening mixed layer due to poor light conditions during the winter months.

Significance of plankton re-entrainment and net biological growth

We can explore further the effect of MLD deepening by incorporating non-zero biological terms in supplementary equation SI2:

$$\frac{dP}{dt} = H(\mu - l)C - W_{eff}C \tag{SI11}$$

The mixed layer average specific growth and loss rates, μ and l, are due to photosynthesis and the sum of zooplankton grazing and phytoplankton mortality, respectively. The sinking term W_{eff} is as defined in Equation SI3. This can be recast in terms of specific rate of biomass accumulation, r:

$$r = \frac{1}{P}\frac{dP}{dt} = (\mu - l) - \frac{W_{eff}}{H} \tag{SI12}$$

where the first RHS term is due to biological growth (r_{bio}), and the second term is due to sinking and re-entrainment (r_{sink}). Biological growth and loss rates are typically in approximate balance (i.e., $\mu \approx l$ and, $|r_{bio}| \ll |\mu|$ and $|r_{bio}| \ll |l|$). Note that the loss of biomass from the mixed layer due to sinking phytoplankton would be larger if re-entrainment is not accounted for, i.e. ($W_{eff} < W_s$), highlighting how deeper winter mixing enhances the retention of biomass.

The northern Labrador Sea chlorophyll inventory (Figs 1 and 3) increases during the period of fall mixed layer deepening (October to February). Assuming the phytoplankton carbon-to-chlorophyll ratio (Θ) to be a constant, we can calculate the positive accumulation rate as:

$$r = \frac{log(\frac{P_{feb}}{P_{oct}})}{\Delta t} = 0.24\, month^{-1} \tag{SI13}$$

The positive accumulation rate ($r > 0$) implies that $\mu > l$, $r_{bio} > r_{sink}$, and $r_{bio} \geq r$ depending on cell size of the population. Hence, the net biological accumulation rate would have to be substantially larger to compensate for sinking losses. For example, for growing plankton with a cell diameter of 50 μm, r_{bio} would have to be about 0.73 month^{-1} averaged over the 4 month fall period. On the other hand, for senescent plankton with a cell diameter of 20 μm, r_{bio} would have to be approximately 0.91 month^{-1}. And if one accounted only for sinking but not particle re-entrainment, r_{bio} would have to be considerably larger. For growing plankton with a cell diameter of 50 μm, r_{bio} would have to be approximately 1.29 month^{-1}, while for senescent plankton with a cell diameter of 20 μm, r_{bio} would have to be about 1.57 month^{-1}.

The fall-winter evolution of mixed layer influences a number of factors that modulate the size of the end of winter phytoplankton population that seeds the large observed

spring bloom surface concentration in April and May following mixed layer shoaling and re-stratification. The plankton sinking flux -W_sC scales with concentration and thus dilution due to deeper mixed layers decreases the magnitude of flux and fractional population loss. The re-entrainment term $\frac{dH}{dt}$C scales with the fall MLD deepening rate and therefore a deeper winter MLD also limits losses from plankton sinking. Based on the scaling arguments provided above, for the northern Labrador Sea the effect of sinking particle flux and re-entrainment is an important contributor, though somewhat smaller than biological effects, and needs to be considered in future projections of climate variability effects on ocean ecosystems.

Impact of photoacclimation on the carbon-to-chlorophyll ratio (Θ)

Our model is constructed around carbon biomass of the phytoplankton stock and we have implicitly assumed a constant Θ. However, the satellite surface chlorophyll data may indeed reflect variations in Θ. To understand this further, we computed the climatological seasonal cycle of Θ for the northern Labrador Sea following [4] as shown below.

We first generate the diffuse attenuation coefficient for PAR (K_d) from K_{490} based on [5] as follows.

$$K_d = 0.0665 + 0.874\,K_{490} - \frac{0.00121}{K_{490}} \tag{SI14}$$

Next we estimate Θ_{DM}, the deep-mixing solution for Θ as

$$\Theta_{DM} = c_1\, e^{\frac{c_2\, PAR^{0.45}}{K_d}} \tag{SI15}$$

where constants $c_1 = 19\ \frac{g\,C}{g\,Chl}$ and $c_2 = 0.038\ m^{-1}$.

Further, we estimate $\Delta\Theta_{SM}$, the shallow mixing correction for Θ as

$$\Delta\Theta_{SM} = \frac{1 + e^{-0.15\,PAR}}{1 + e^{-3\,I_{ml}}} \tag{SI16}$$

where I_{ml}, the median light level in a well-mixed surface layer is given by

$$I_{ml} = PAR\, e^{-0.5\,K_d\,MLD} \tag{SI17}$$

Now, having computed Θ_{DM} and $\Delta\Theta_{SM}$, Θ is estimated as their product. Based on this calculation, the values of Θ from February to October are: 22.2, 23.6, 21.1, 20.7, 39.4,40.6, 35.0, 26.5 and 22.0. Note that between October and March, the period of interest in our study, Θ changes by approximately 7% and does not vary appreciably over the fall and winter months, especially compared to the summer months of June and July where it nearly doubles compared to its winter values. Based on this analysis, we think it is reasonable to assume a constant value for Θ and ignore the effects of photoacclimation in our study.

Analysis of various factors contributing to stratification changes in the northern Labrador Sea

To ensure that Arctic sea ice melt is the primary reason for the projected decrease in wintertime convection in the Labrador Sea, we examined other factors that may also impact MLD (S3 Fig). First, we considered impacts from dynamical changes induced by surface winds. There is a weak negative trend in Ekman pumping, suggesting that long term changes in surface winds tend to reduce upwelling and deepen the thermocline.

Hence, changes in surface winds over the northern Labrador Sea cannot be responsible for the reduction in MLD. Next, we considered changes in surface density. We find a significant decrease in surface density with time, and the enhanced stratification likely caused the reduction in MLD. Further, to understand the relative roles of temperature and salinity in density changes, we computed the density time series by varying salinity with a constant initial temperature and vice versa. While temperature tends to cause a weak increase in surface density, salinity acts to reduce density strongly. Based on this, it is clear that changes in surface density in the northern Labrador Sea were primarily driven by those in surface salinity. Furthermore, to understand the cause behind changes in surface salinity, we looked at precipitation changes. There is a significant negative trend in rainfall over the northern Labrador Sea, revealing that long term changes in precipitation cannot have caused the substantial upper-ocean freshening in this region. With these various factors exhausted, the only possibility that remains is advection of freshwater from the Arctic region.

Supporting Information References

1. Behrenfeld MJ. Abandoning Sverdrup's critical depth hypothesis on phytoplankton blooms. Ecology. 2010;91(4):977–989.

2. D'Asaro EA. Convection and the seeding of the North Atlantic bloom. Journal of Marine Systems. 2008;69(3):233–237.

3. Smayda TJ. The suspension and sinking of phytoplankton in the sea. Oceanography and Marine Biology. 1970;8.

4. Behrenfeld MJ, O'Malley RT, Boss ES, Westberry TK, Graff JR, Halsey KH, et al. Revaluating ocean warming impacts on global phytoplankton. Nature Climate Change. 2016;6(3):323–330.

5. Morel A, Huot Y, Gentili B, Werdell PJ, Hooker SB, Franz BA. Examining the consistency of products derived from various ocean color sensors in open ocean (Case 1) waters in the perspective of a multi-sensor approach. Remote Sensing of Environment. 2007;111(1):69–88.
