# Supplementary figures and images for: Linking deep convection and phytoplankton blooms in the northern Labrador Sea in a changing climate
Source: PLoS One. 2018 Jan 25;13(1):e0191509. doi: 10.1371/journal.pone.0191509 (PMC5784959; doi:10.1371/journal.pone.0191509)

A)

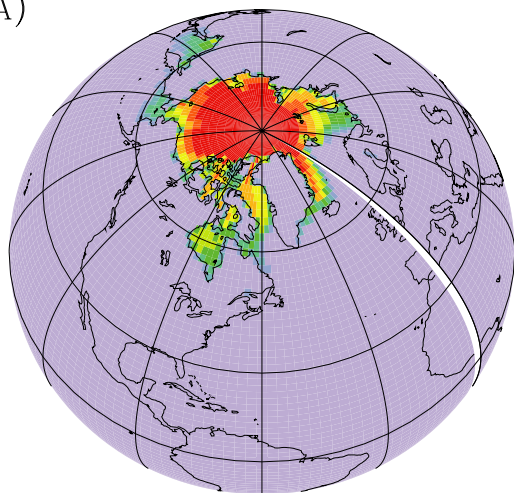

Ice Fraction (1990–2005)

B)

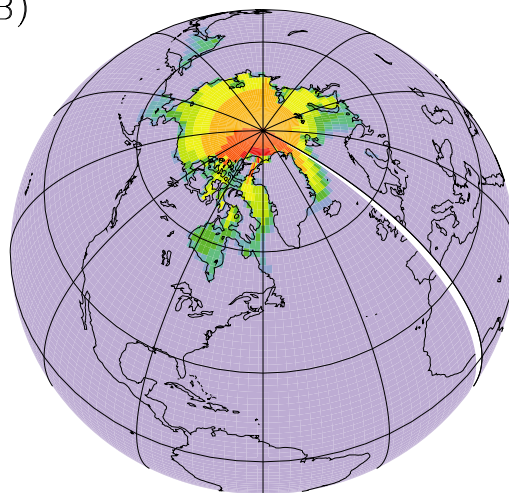

Ice Fraction (2020–2029)

C)

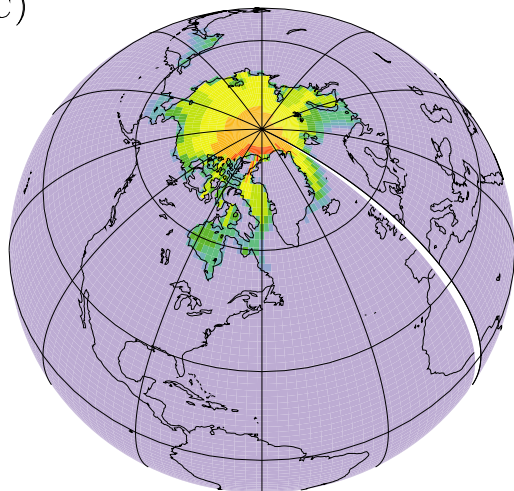

Ice Fraction (2030–2039)

D)

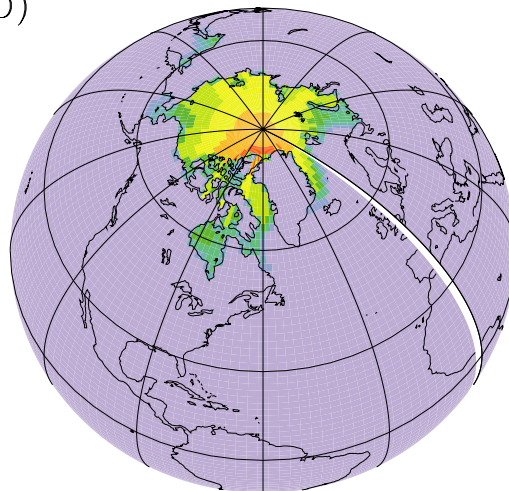

Ice Fraction (2040–2049)

Supplement: S1 Fig — A: Mean ice-fraction averaged over the years 1990-2005. Mean ice-fraction projected under the RCP 4.5 scenario averaged over the years B: 2020-2029 C: 2030-2039 D: 2040-2049. (PDF) [file pone.0191509.s002.pdf]

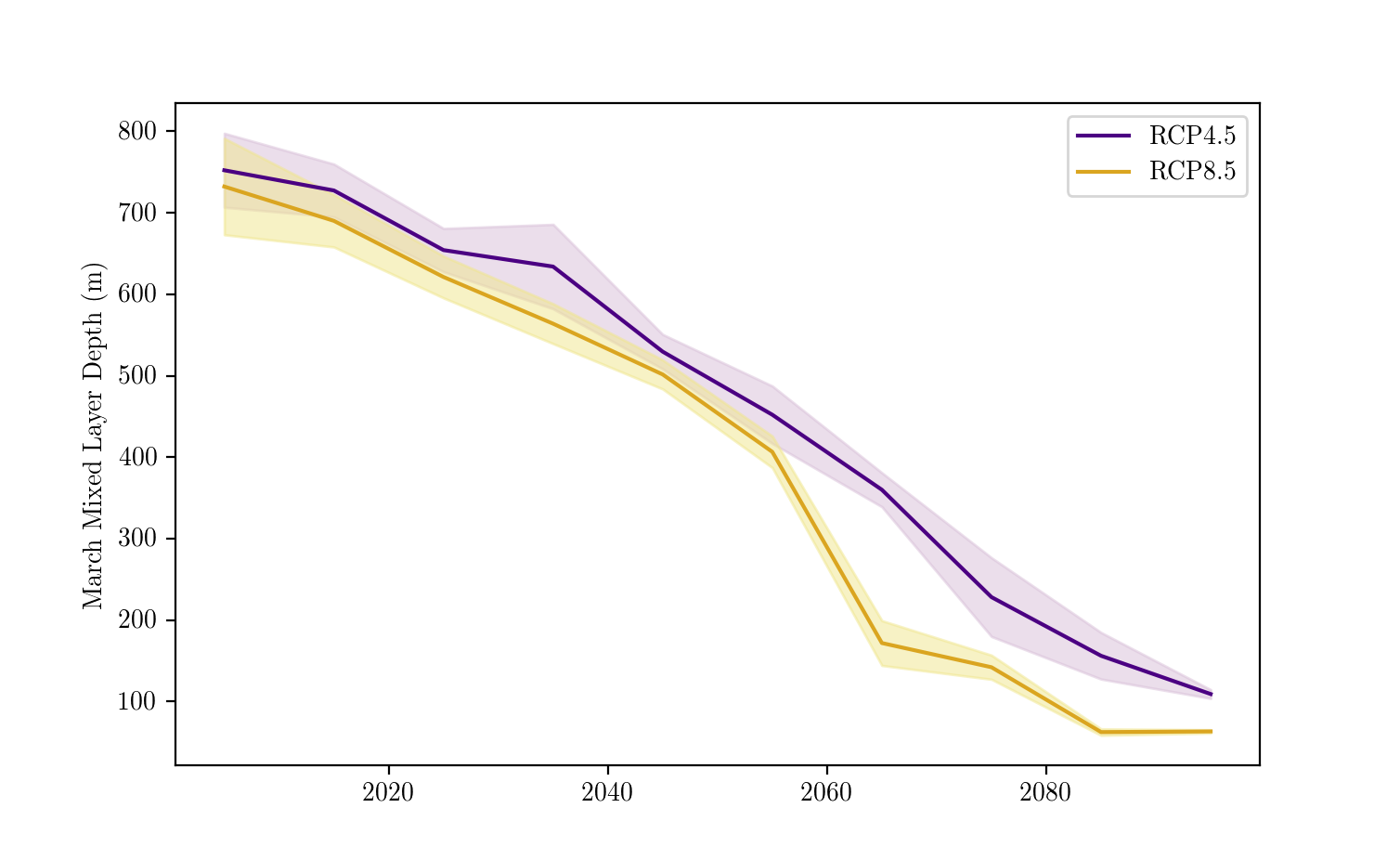

Supplement: S2 Fig — Projected changes in March MLD (m) conditions in the northern Labrador Sea under the RCP 4.5 (purple) and the RCP 8.5 (yellow) scenarios. The ensemble mean is indicated by the thick line, while the shading around the line represents the spread among the ensemble members. (PNG) [file pone.0191509.s003.png]
